# Developing research questions in conversation with the literature: operationalization & tool support

**DOI:** 10.1007/s10664-022-10204-8

**Published:** 2022-09-20

**Authors:** Oscar Díaz, Jeremías P. Contell

**Affiliations:** ONEKIN Research Group, University of the Basque Country (UPV/EHU), San Sebastian, Spain

**Keywords:** Research question, Literature review, Inductive Top-Down Theorizing, Reference management systems

## Abstract

Empirical Software Engineering rests on the understanding of practical problems and their solution counterparts. Frequently, solutions are not absolute but relative to the context where the problem is observed. This tends to imply that the solution and the problem unveil gradually together, and hence, researchers are not always in the position to state the research question (RQ) at the onset. Like software engineers when facing blurred requirements, researchers might not be familiar enough with the problem in the early phases of a research to properly scope their RQs (hereafter referred to as RQ Scoping). Here, the literature may play the role of the stakeholders in Agile methods: keeping the focus on the aspects that are essential (vs. accidental) of the RQ. Informed by Inductive Top-Down Theorizing, this article acknowledges RQ Scoping as iterative and incremental, entailing a conversation between the experimental work and literature reviewing. Yet, for literature reviewing to become “Agile” it is not only required to be driven by the RQ but also to have tool support. Tools might bring transparency and traceability, both factors especially welcome in a scenario characterized by testing (is my RQ relevant?) and adjustment (how can I make my RQ relevant?). Specifically, the advent of the RQ in close relationship with the literature advises for “Agile” literature reviewing to be conducted at the place where the literature is naturally kept: the Reference Management System (e.g., Mendeley). This article introduces the theoretical underpinnings, design principles, proof of concept and evaluation for FRAMEndeley, a Mendeley-integrated utility for RQ Scoping.

## Introduction

Empirical Software Engineering (ESE) is gaining momentum as research is headed towards an evidence-based understanding of practical problems and their solution counterparts (Basili et al. 1986). “Empirical” stands for being verifiable by observation or experience rather than theory or pure logic. Therefore, the context in which this observation or experience is acquired becomes a critical ingredient of ESE. Here, solutions are not absolute but relative to the context where the problem is observed. How one frames the problem shapes its solution, and the problem and solution are in this way intrinsically related. As a result, in order to handle ill-structured problems, one must formulate problems as a core aspect of the investigation (Nielsen and Persson 2016). This positions a big part of ESE research as problem-solving, and the object of study as “an artifact in a context” (Wieringa 2014). Here, research faces *design questions*, i.e., what would a solution look like for the problem at hand under a given context? Accordingly, the description of design questions moves beyond the problem space (Context & Goal) to also include the solution space (Artifact & Requirement) (Wieringa 2014). Indeed, Dybå et al. (2012, p. 25) state that “the characteristics of research evidence as an interactive phenomenon, challenge the traditional notions of empirical SE, suggesting a view of the relationship between evidence and context as a process that emerges and changes through time and space”.

Deciding on the design question is likely the most important choice that a researcher makes, as it is a decision on what deserves scientific investigation. The goodness of this decision is to be affected by the process being followed (Rai 2017). This is particularly challenging in ESE where problems and solutions unveil gradually together. This raises the “Goldilocks issue” whereby researchers struggle with scoping decisions to avoid excessive or marginal scope (Rai 2017). We term *Research Question Scoping* (RQ Scoping) the process of iteratively and gradually formulating and re-formulating the concepts that underpin the characterization of problems & solutions, that eventually ends up into a design question. RQ Scoping extends the traditional notion of problem formulation (Rai 2017) to also include the envisaged solution.

RQ Scoping is difficult (Hassan 2017). Difficulty stems from its abductive nature. Abductive Reasoning starts with a set of observations (i.e., empirical data), and then, seeks to find the simplest and most likely explanations for the observations: the hypotheses (Walton 2014). This starts an exploration journey, where the recently acquired hypotheses are put to the test, more data are generated, and eventually, a new hypothesis emerges.

Yet, it is unclear how hypothesis generation is conducted. Different authors highlight the importance of the literature in this process (Shepherd and Sutcliffe 2011; Wolfswinkel et al. 2013; Hoda 2021). In the ESE field, Eisenhardt (1989, p. 544) states that to move from case study data to a significant contribution, literature revision should be conducted in terms of the “comparison of the emergent concepts, theory, or hypotheses with the extant literature”. Accordingly, coming up with a RQ (i.e., RQ Scoping) is acknowledged as iterative and incremental, entailing an interplay between the experimental work (e.g., artifact development) and literature reviewing. For the sake of understanding, we can draw a parallel with Agile software development (Beck et al. 2001). In this comparison, the application requirements and the stakeholders find their counterparts in the RQ and the literature, respectively.

Similitude with Agile is four-fold. First, like software engineers, researchers might not be familiar enough with the problem in the early phases of a research to properly scope their RQs. Agile resorts to stakeholders to keep the development team focused on the solution’s intended goals. Likewise, the literature plays the role of the stakeholders in keeping the focus on the aspects that are essential (vs. accidental) of the RQ. Second, Agile development advances iteratively and gradually through sprints. Likewise, coming up with a RQ is not a one-time activity but the RQ unveils as new or refined insights emerge. Third, Agile aims at an early and continuous delivery of working software. Likewise, RQ Scoping aims at producing successive versions of the RQs that “work”, i.e., that promote reflection and analysis through Comparative Thinking. Fourth, Agile is not about tools, but tools were key for Agile to become mainstream (e.g., Jira). Smooth adoption of RQ Scoping also rests on the existence of tools that support iterative and long-lasting reviewing sprints. Besides performance, tools bring transparency (the quality of being done in an open way) and traceability (the quality of having an origin that may be found or followed), both factors especially welcome in a scenario characterized by testing (is my RQ relevant?) and adjustment (how can I make my RQ relevant?).

RQ Scoping is particularly relevant for novice researchers. To many postgraduate students, finding the “right” direction in the literature maze is a particularly grueling experience (Kwan 2008). Novice researchers need a greater level of structure when it comes to designing and conducting their research. Klopper et al. (2007, p. 272) outline a too familiar scenario when talking about postgraduate students: “they cast about collecting data with no defined problem statement from which they extract keywords to serve as filter for the identification of relevant literature. They read each reference in detail rather than using abstracts and summaries to establish relevance, and they start summarizing the literature with no plan in mind, and end up with a document without a proper layout, showing no coherence and progression”. Hence, postgraduate students will be particularly our target audience.

In short, if RQ Scoping is the aim, then “Agile Literature Reviewing” might well be the means. Specifically, the advent of the RQ in close relationship with the literature advises for RQ Scoping to be conducted at the place where the literature is naturally kept: the Reference Management System (RMS). RMSs like Mendeley, Zotero or EndNote are nowadays common among researchers (Fitzgibbons and Meert 2010). Rather than providing a separated tool, we make a case for RQ Scoping to be integrated within RMSs. Accordingly, we introduce this work’s research question:

*In ESE research, how would RQ Scoping be operationalized as a RMS utility with a focus on postgraduate students?*

In addressing this question, we contribute to the existing body of literature by:

- operationalizing RQ Scoping as an abductive effort (Section 3),
- introducing design principles for the development of RQ Scoping utilities (Section 5), and
- developing and evaluating a purposeful artifact, *FRAMEndeley*, that supports RQ Scoping for Mendeley as the RMS (Sections 6 and 7).

We start by providing background on design questions and literature exploration.

## On Design Questions

A Research Question (RQ) is an “answerable inquiry into a specific concern or issue” (Studycom 2013). The relevance of RQs is emphasized in the literature. Recker(2013, p. 27) regards RQs as “the fundamental cornerstone around which your whole doctoral research revolves and evolves” while Alvesson and Sandberg (2013, p. 11) point out that “without posing questions it is not possible to develop our knowledge about a particular subject”. Thuan et al. (2019) distinguish three roles for research questions: 1) defining the scope of the research, 2) directing the research process, and 3), positioning the contributions. These roles apply no matter the scientific discipline. Hence, what is specific about ESE?

When compared with other scientific fields, a relevant part of ESE is problem-solving, i.e., designing artifacts as solutions to mitigate a problem. Unlike *knowledge questions* that focus on “the what”, *design questions* move to “the how” by producing prescriptive knowledge to apply in context (Wieringa 2014). This context is normally socio-technical. Here, the interplay of settings and techniques influences the functionality and usage of the resulting artifact (Baxter and Sommerville 2011). Hence, problem-solving often implies the existence of artifacts as interventions that are deployed in a given context in the search for some utility (Venable et al. 2016). Accordingly, design questions profile both the problem space (Context & Goal) and the solution space (Artifact & Requirement). To this end, Wieringa (2014) introduces the following template for design questions:

How to <(re)design an **Artifact**> that satisfies <some **Requirements**> in order for <stakeholders to achieve some **Goals**> in <a problem **Context**>?

We will refer to this template as CGAR (pronounced like “cigar”).^1^ Wieringa’s template signifies the entanglement between problem and solution, critical to ESE. As Wieringa himself is quick to point out, researchers are hardly in the position of fully filling up the CGAR template at the onset of the research (Wieringa 2014). Even more, there exists the risk of biasing the RQ efforts towards solving the problem rather than defining it (Iivari 2007). This vindicates a shift in focus from the result (i.e., the RQ) to the process (i.e., RQ Scoping). But, how does this process look? This is when the literature comes in.

## Literature Reviewing: Themes & Variations

Researchers approach the literature with different purposes. This section frames RQ Scoping within other processes where the literature also plays a key role: Systematic Literature Reviews, Schema Development, Grounded Theory, and Abductive Reasoning.

### Systematic Literature Reviews

Systematic Literature Reviews (SLRs) aim at coming up with the “map” of a profession, theory, or practice, normally at a given point in time (Kitchenham et al. 2011). It is then a one-time effort that usually takes several months but time bounded. Abundant documentation exists on how to conduct this review systematically for several fields: software engineering (Kitchenham et al. 2015), health sciences (Fink 2019), or social sciences (Petticrew and Roberts 2006). The process starts with a RQ that guides the elaboration of the “search string”, i.e. the query to be posed to the bibliographical databases. This search string is tuned during the pilot study but tends to be kept quite stable once data extraction begins (Felizardo et al. 2017). Alternatively, “the trial-and-error” search is being proposed to initially test and develop search queries, e.g., by determining which keywords might (not) generate useful results (Kuhrmann et al. 2017). Nevertheless, the challenge is not so much about search string calibration but completeness. This explains the use of snowballing techniques to ensure an appropriate coverage (Wohlin 2014). Collected studies go through the sieve of the inclusion and exclusion criteria. These criteria tend to be kept stable throughout the review, and they are generally related with the quality of the primary study. For our purposes, we notice that SLRs focus on completeness, systematicity of the process, and reproducibility of the results (Hoda 2021).

### Schema Development

Schema Development aims at coming up with a schema, i.e., a cognitive or behavior pattern that organizes categories of information and their interconnections (DiMaggio 1997). People use schemata to organize *existing* knowledge and provide a framework for future understanding. Rather than rumbling across the literature, researchers pursuing schema development focus on the most influential articles from where to extract main constructs. Instead of skimming and classifying all articles, and next, selecting the ones that require thorough reading, proponents of this approach advocate for reversing these phases (Wagner et al. 2020). Unlike SLRs, Schema Development puts the emphasis on identifying the (few) most influential articles for readers to acquire a better understanding of the foundations of a domain. Schema Development filters articles based on their influence. To identify relevant articles, methodologists provide heuristics. For instance, in the area of management, Blumberg et al. (2014) propose heuristics based on citations, recency or uniqueness. The rest of the articles are merely skimmed. Schema Development’s theoretical underpinnings are based on the Schemata Theory, which describes the underlying cognitive comprehension processes as associating new pieces of information with prior knowledge (Anderson 2017). For our purposes, we notice that Schema Development looks not so much for completeness but for gradually cherry-picking the most influential articles.

### Grounded Theory

Common in Qualitative Data Analysis (QDA), Grounded Theory (GT) is a method of inductively generating theory from data (Corbin and Strauss 2014). Here, concepts are said to “emerge” from the data. These concepts are tagged with *codes* that abbreviate these concepts. As more data are collected and re-reviewed, codes can be grouped into higher-level *themes* and next, into *categories*. These categories set the basis for the conceptual framework that might give rise to a theory where a causal relationship among concepts is hypothesized (Braun and Clarke 2006). Here, the data take the form of interview transcripts, documents, or field notes that are obtained from a sample population. The objective of sampling from a population is to select a subset of units from the population that is representative of. Hence, sampling is not about achieving numerical significance, but representativeness, i.e., being able to contribute meaning to, illuminate, and, in some circumstances, explain the phenomenon under investigation. Important to note, if sampling is said “theoretical”, then the incipient theory is used to guide which people to select for the next “transcript crop”. Essentially, in GT, theoretical sampling is directed toward expanding on and profiling categories. Theoretical sampling is not about merely extending the data set (Timonen et al. 2018).

What is then the role of literature in GT? Although initially GT builds theories by uniquely resorting to empirical data without engaging with existing literature before themes and codes have been fully theorized (aka Glaserian GT), later amendments consider that initial data may lead the researcher beyond empirical observation to engage with the literature in the search for novel explanations and theory constructs (aka Constructivist GT) (Stol et al. 2016). In *theoretical sampling*, the researcher identifies further data sources based on gaps in the emerging theory to further explore concepts. This is where the literature might serve to cast doubt on or confirm current insights, helping researchers come up with new hypotheses to be validated *empirically* (Charmaz 2014), e.g., conducting additional interviews. In this scenario, the literature informs but it is not the subject of coding. Alternatively, GT has also been proposed as a method for literature review (Wolfswinkel et al. 2013). In this mode, the literature *is* the data source. Instead of being deductively derived beforehand, concepts are derived from the literature (Wolfswinkel et al. 2013).

### Abductive Reasoning

Abductive Reasoning entails three main steps (Walton 2014). It starts with a set of observations. Next, it seeks to find the simplest and most likely explanations for these observations, i.e., the hypothesis (induction). And finally, this hypothesis is to be confirmed by a new series of observations (deduction). This *modus operandi* characterizes the detective’s work when collecting evidence (i.e., empirical data) from the scene of a crime in ascertaining the suspects (i.e., the hypothesis). Besides empirical data, researchers have an additional source of information: the literature. The researcher’s attention can be focused by his knowledge and scholarly context (e.g., derived from empirical observations and previous experiences) but also informed by the literature. The weight that each of these informants (i.e., empirical data vs. the literature) have on the abductive process results in different degrees of “inductive-ness” and “deductive-ness”. When it comes to literature reviewing, the main driver is now affinity, i.e., finding articles that might challenge the researcher’s hypothesis. Here, the literature review does not aim at covering all articles about a theme nor finding the most influential ones but rather, challenging the researcher’s assumptions. Unlike some SLR interventions, the goal is not so much “gap spotting” but problematizing the literature.

In Social Sciences, an early attempt to apply this approach is the Inductive Top-Down Theorizing (ITDT) model (Shepherd and Sutcliffe 2011). More recently, the Socio-Technical Grounded Theory (STGT) applies similar insights as a variation of GT tuned for socio-technical research (Hoda 2021). STGT also advocates for Abductive Reasoning by interleaving “rounds of basic data collection and analysis, emergent or structured mode of theory development through advanced data collection, analysis, and theory development procedures” (Hoda 2021, p. 7). STGT distinguishes two key stages. The Basic stage aims at obtaining “a few strong categories and preliminary hypotheses”. The Advanced stage pursues theory development. RQ Scoping can be framed within the Basic Stage. Yet, neither ITDT nor STGT operationalize the elicitation of “a few strong categories and preliminary hypotheses”.

### RQ Scoping

Broadly, RQ Scoping can be described as Abductive Reasoning “in-the-small”. RQ Scoping is “small”: it does not aim at coming up with a full-fledged theory but just a RQ, i.e., a question that merits scientific investigation. To this end, RQ Scoping sticks to Abductive Reasoning. Broadly, the RQ plays the role of a hypothesis about the originality of the research. The literature is the Acid Test. During the test, researchers follow Comparative Thinking, which can be regarded as the counterpart of GT’s “constant comparison” *motto*. Comparative Thinking seeks complementarities or dissimilarities with related work (Mussweiler and Posten 2012; Marzano et al. 2001). Seeking complementarities refers to positioning findings to the extant theoretical stream (aka synergistic positioning) (Ridder et al. 2014). This favors knowledge accumulation by allowing researchers to elaborate a phenomenon in greater detail. By contrast, seeking dissimilarities profiles one’s work in contrast with theories that diverge in either the problem being tackled or the solution being proposed (aka antagonistic positioning) (Ridder et al. 2014). **The aim is to fine-tune the hypothesis (i.e., the RQ) by challenging the researcher’s assumptions.**

In this setting, literature reviewing is recurrent and contingent. The RQ is tentative. The CGAR template might still be in an embryonic state with a blurring understanding of the key factors that capture the essence (in contrast with the accident) of the Context, Goal, Artifact, and Requirements. In this scenario, literature reviewing might not afford to be “systematic”. An Agile approach might be a better fit. In this comparison, the application requirements and the stakeholders find their counterparts in the RQ and the literature, respectively. Like software engineers, researchers might not be familiar enough with the problem in the early phases of a research to properly scope their RQs (the counterpart of the application requirements). To combat this, Agile resorts to an iterative and gradual development process (i.e., the sprints). Likewise, RQ Scoping becomes “agile” insofar as the RQ unveils gradually and iteratively as confronting the literature by means of Comparative Thinking.

**Fig. 1:**
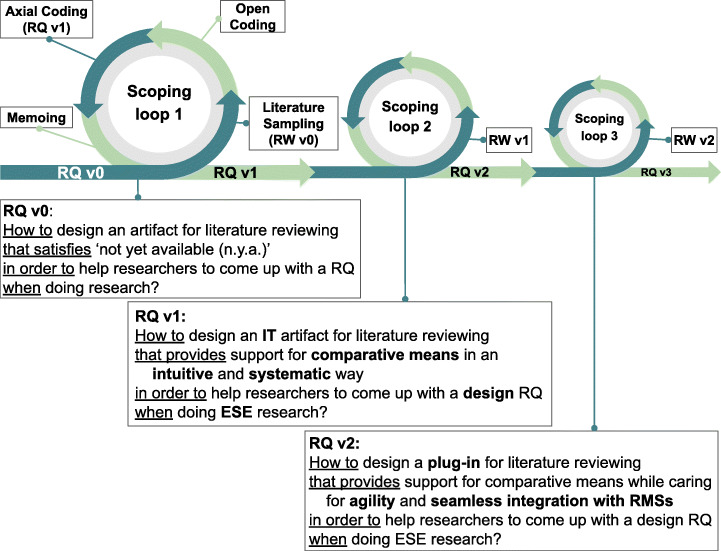
RQ Scoping: literature sampling and RQ evolve in tandem. In RQ formulations, bold font stands for the difference with the previous version

Figure 1 abounds in this similitude between software development and RQ Scoping. To a certain extent, SLRs follow a waterfall model: the RQ is generally set at the onset, after some pilot study (Felizardo and Carver 2020; Wolfswinkel et al. 2013). Alternatively, RQ Scoping starts with an often incomplete RQ v0 (i.e., not all CGAR constructs are yet decided). This RQ often delivers an abundant collection of related work (RW v0) whose reading helps better tune the research question RQ v1, and this starts the cycle again. The RQ (as the software requirements) is iteratively unfolded as confronted with the literature (as the stakeholder counterpart). In Agile, new requirements might emerge as stakeholders gain insights about the software. Likewise, the RQ is not free from unexpected changes if significant literature shows up. As the process progresses, the RQ is better profiled, hopefully leading to smaller RW sets (depicted through smaller sprints in Fig. 1). Next, we describe our own journey in coming up with the RQ of this very work.

## RQ Scoping at Work

This section introduces the RQ of this work. Yet, it is not the destination (RQ) but the journey (RQ Scoping) that matters. Though a bit premature in introducing some concerns that are developed later in the article, the narrative of this section illustrates the sort of journey we want to give support to. Throughout this journey, different “sprints” were conducted with the set of articles ranging from thirty to eleven, accounting in the end for around eighty articles consulted.

The journey may start with an intuition, perhaps a gut feeling from the student’s supervisor. This is RQ v0 (see Fig. 1). It is not a bad beginning. It sets the Context (i.e., doing research), the Goal (i.e., coming up with a RQ), and the Artifact (an artifact for literature reviewing) while Requirement is left undefined. When reading the literature for the purpose of RQ Scoping, the student starts by identifying the C, G, A and R out of text fragments from the literature (aka excerpts), i.e., open coding. Of course, finding codes might not be trivial. Neither is it a one-time activity. Chances are the student needs to change the coding schema as new studies come up. Table 1 is the outcome of a GT effort that expanded for over a year. Next, we outline a close-to-reality scoping process. The framing of the problem (C, G) and the solution (A, R) unfold jointly and gradually throughout.

**Table 1:** Literature Reviewing in CGAR-centric RQ Scoping: studies are regarded as cases where to identified Context, Goal, Artifact and Requirement

Work	Context	Goal	Artifact		Requirements	
			Type	Architecture	Functional	Non-functional
Klopper et al. (2007)	SS	gap spotting	method	n.a.	comparative means	n.a.
Benmerikhi and Leuridan (2015)	SS	RQ Scoping	method	n.a.	empirical saturation	n.a.
					theoretical saturation	
Boell and Cecez-Kecmanovic (2014)	IS	theorizing	method	n.a.	note-keeping	n.a.
					snowballing	
Wolfswinkel et al. (2013)	IS	synthesizing	method	n.a.	code-book	n.a.
					note-keeping	
Wagner et al. (2020)	IS	schema extraction	method & tool	standalone	article prioritization	accuracy
Fernández-Sáez et al. (2010)	SE	synthesizing	tool	standalone	literature search	systematicity
					comparative means	scalability
					reporting	
Bowes et al. (2012)	SE	synthesizing	tool	standalone	literature search	systematicity
					data extraction forms	scalability
					reporting	
Molléri and Benitti (2015)	SE	synthesizing	tool	standalone	literature search	systematicity
					data extraction forms	scalability
					comparative means	
					reporting	
Fabbri et al. (2016)	SE	synthesizing	tool	standalone	literature search	systematicity
					data extraction forms	scalability
					reporting	
Diaz et al. (2017)	IS	schema extraction	tool	plug-in	color-coding	systematicity
This work	SE	RQ Scoping	method & tool	plug-in	literature search	agility
					color-coding	seamless integration
					code-book	
					comparative means	
					theoretical sampling	

*On the problem side*, the student notices that excerpts introduce different ways for helping with the Goal: “to come up with a RQ”. She abstracts away from the wording of the excerpts, and introduces three codes: *synthesizing, gap spotting* and *schema extraction*. Later on, when reviewing studies from Social Sciences, the student unveils a new Goal: *theorizing* for supporting nascent theories through Abductive Reasoning (e.g., Shepherd and Sutcliffe’s 2011 ITDT). The knowledge field is different—Social Sciences (SS) vs. Software Engineering (SE) vs. Information Systems (IS)—but, initially this difference looked accidental. On second thoughts, however, RQs in SS (i.e., knowledge questions) reveal themselves as different from design questions as observed by Wieringa. So we notice it in the RQ v1, which now refers to *design* RQ.

*On the solution side*, the student comes across with Webster and Watson’s (2002) Concept Matrices, and Klopper et al.’s (2007) Comparative Matrices. Using Klopper et al.’s (2007, p. 268) own description: “articles are listed below one another in the leftmost column of the matrix, with concepts A, B, C, D ... being listed at the head of subsequent columns so that for each article the presence of a particular concept can be marked with a right tick in the appropriate cell of the matrix. The use of such a concept matrix enables the researcher to directly establish at a glance, which articles deal with a particular research theme, enabling the researcher to explicitly identify, classify and assess facts thematically, rather than infer them indirectly from memory of articles read in isolation of one another”. She then realizes the importance of “Comparative Thinking” as a means for assumption challenging and its operationalization through comparative matrices. For this strategy, the student values its intuitiveness (the quality of being perceived directly by intuition without rational thought) and systematicity (the quality of being methodical, regular and orderly). Hence, she decides to abstract from this realization (i.e., “comparative matrix”) through the code “comparative means”. The code stands for a functional Requirement to signify resources that foster Comparative Thinking. Next, the student tries Klopper et al.’s comparative matrices to get some empirical data. The hands-on experience works fine for up to ten studies but above this number, matrices did not scale so well, more to the point, if the article set is in flux. If conducted manually, chances are comparative matrices become time and error-prone. Software assistance is needed. The artifact needs to be qualified: “artifact” becomes “IT artifact”. This results in RQ v1 (see Fig. 1).

Hereafter, the student slides towards the solution realm. Dedicated tools exist for QDA and SLRs.^2^ For QDA, notorious examples include NVivo®; (QSR International Pty Ltd), MAXQDA (VERBI GmbH), and ATLAS.ti®; (Scientific Software Development GmbH). QDA tools excel at coding while SLR tools stand out at protocol assurance and collaboration. The student tries some of these tools to obtain some empirical data. At this time, new functional requirements are identified: “color-coding”, “code-book”, “snowballing” or “theoretical sampling” to name a few. The latter becomes particularly relevant since it underpins one of the most distinctive features of RQ Scoping: the co-evolution of the RQ and the focus of the literature search. The student notices that “theoretical sampling” is often found in QDA tools, while it is almost absent in SLR tools, being not even a main demand among SLR practitioners (Al-Zubidy and Carver 2019).^3^ The student observes that tools differ in their business model: proprietary software vs. open source. The student feels this is accidental. She records the observation but no code is generated. By looking at the tools’ documentation, the student notices authors stress distinct concerns. QDA tools attend to scalability in pursuit of coping with a potentially large number of transcripts (or other qualitative data). SLR tools put the focus on “traceability” to support replicability in data extraction, which in turn impacts “trustworthiness”. This is particularly relevant for both QDA and SLRs since their outcomes aim to inform other researchers. On the contrary, the student realizes that RQ Scoping involves fewer articles at a time, mainly for self-consumption, at best to be shared with her supervisor. Therefore, “scalability” is not such a stringent demand but the need for an agile (de)construction of themes becomes more relevant. Notice that “agility” is not a function like “color-coding”, but it indicates how the function should be conducted. In other words, “color-coding” does not pertain to the same semantic space as “agility”. This results in codes to be split along two (sub)themes: Functional and Non-Functional. This might look like an obvious distinction. Notice however that the emergence of “Non-functional” as a sub-theme is grounded not only on the existence of excerpts but on the fact that this theme is conjectured as “essential” to discriminate the RQ at hand. In RQ Scoping, the researcher strives to come up with, not just any theme, but themes that single out the RQ with respect to existing works. Lastly, the introduction of non-functional requirements may impact the architecture of the artifact. The student notices that QDA and SLRs tend to be supported through dedicated tools. She wonders whether a standalone tool is also the best option for RQ Scoping. Some experimental work is due. In QDA, most transcripts are read and coded. In RQ Scoping, most articles are scanned but few are coded. Or they might be coded and discarded at a later stage. Or they need to be re-coded as a result of the emergence of a new theme. This close and *continuous* involvement with the literature grounds the case for RQ Scoping to be seamlessly integrated with RMSs. Architecturally wise, this calls for a plug-in rather than a standalone tool. This is felt discriminant enough to vindicate a new sub-theme: “Architecture”. This leads to RQ v2.

In the end, around eighty studies have been read *over a year*. Table 1 lists the closest to our intervention (i.e., RW v2). This number is not far from the amount of primary studies considered in SLRs. Differences mainly stem from both *when* they are read (a longer time span) and *how* they are read (contrasting with one’s work). The latter is especially relevant. As an abductive process, RQ Scoping intermingles induction (looking for themes via GT) and deduction (looking for hypotheses via Comparative Thinking). GT accounts for inductive reasoning, i.e., it lets the salient concepts arise from the literature. For instance, when looking at excerpts describing Artifacts, concepts such as “Type”, “Architecture” or “Approach” arise. We do not know which of these concepts will become essential (vs. accidental) to profile our work. All induced concepts matter. You can try to see the generality of these concepts by using them to characterize additional works (deductive reasoning). This is the prevalent way in literature synthesis. Yet, RQ Scoping goes a step forward. Existing concepts, or better said, the way these concepts are combined in the RQ template, should be opposed to existing works. Broadly, the RQ plays the role of a hypothesis about the originality of the work. The literature is the Acid Test. During the test, some concepts might go away (e.g., “Approach” is considered accidental, not discriminant enough) while brand-new concepts are introduced (e.g., “Architecture”). Here, “plug-in” does not arise through coding (induction) but as a sort of “gut feeling” (most likely supported by some co-existing empirical experimentation); “plug-in” is a bet the researcher makes in the pursuit of originality and relevance. This bet is put to the test in “the roulette of the literature”: has RQ v2 been already investigated? Is there someone else that resorts to plug-ins for QDA or SLRs?

In short, Systematic Literature Reviews and Agile Literature Reviews are two means with different ends. In Systematic Literature Reviews, the output is the answer to the RQ of the SLR, where replicability and thoroughness are critical for gaining trust from the target audience: the community. By contrast, in Agile Literature Reviews, the output is the RQ itself, and agility and interoperability with RMSs are critical for being adopted by the target audience: the reviewer herself. We can then conclude that RQ Scoping exhibits some specifics, distinctive enough, to vindicate the existence of dedicated tools: the Scoping utilities.

## Design Principles for Scoping Utilities

This section generalizes the Design Principles (DPs) derived from the experience of leveraging a RMS (i.e., Mendeley) with RQ Scoping capabilities (see Section 6). Design Principles reflect knowledge of both IT and human behavior. Accordingly, a design principle should provide cues about the effect (RQ Scoping activity made possible), the cause (affordability brought about by the tool), and the context where this cause can be expected to yield the effect for the target audience (Gregor et al. 2020). For our purposes, the context is that of problem-solving ESE research, conducted primarily by novice researchers that keep their bibliography into RMSs.

For the purpose of this work, a Scoping utility is an IT artifact that helps in coming up with a RQ in close relationship with the literature. We advocate for RQs to be described in terms of themes. A “theme” is defined by DeSantis and Ugarriza (2000, p. 362) as “an abstract entity that brings meaning and identity to a recurrent experience and its variant manifestations”. As themes, “Context”, “Goal”, “Artifact” and “Requirements” realize “abstract entities” that represent “recurrent experiences” in capturing design questions. However, these themes might turn too “abstract” during Comparative Thinking. Here, the researcher needs to place her work *vis-à-vis* existing works to signify a distinctive contribution to the field, and this might require the introduction of finer-grained themes that capture the originality of the RQ. More formally,

Let T be a set of meaningful themes that characterize both the problem space and the solution space. Let C_*T*_ be the set of meaningful codes for theme T, including code “n.y.a.” (not yet available). A RQ is a set of pairs (t, c) with t∈T ∧ c∈*C*_*T*_ where each pair positions one’s research along the design dimensions captured through T.

By characterizing a RQ as a set of pairs (t, c), RQ Scoping becomes a GT endeavor. Operationalization wise, RQ Scoping involves the joint and gradual evolution of two variables: the *Research Question* variable (*RQ*) as “the theory”, and the *Related Work* variable (*RW* ) as “the ground data”. Next, we introduce a set of mechanisms for making these two variables evolve in unison (see Table 2).

### Initializing the Variables: RQ and RW

The RQ variable starts off from Wieringa’s (2014) template: CGAR is used as a head-start for RQ definition. Different entry points can be envisaged for elaborating the CGAR template. Researchers coming from industry might know about the problem (CG) from the onset, and then put the focus on the AR. Alternatively, researchers might attempt to appropriate an existing technical solution (AR) to an application scenario different from the original one. Finally, it is not odd that the researcher has no real experience on neither the problem nor the solution. Here, problems and solutions unveil simultaneously. The student’s supervisor might facilitate a set of “seminal articles”. These articles help find preliminary codes to characterize the RQ. This resembles the pilot stage in SLR protocols. In general, researchers might provide tentative values, necessarily vague, to fill up the CGAR template. At this point, code “not yet available” is a possible option.

(DP1) **Provide the RMS with** a CGAR template **in order for researchers to** set CGAR as the departing themes for RQ definition.

The RW variable keeps the articles that help scope the RQ through CGAR-based coding. Yet, not all articles are worth coding. Articles are first scanned, and only if judged of interest, are then moved to the coding stage. If the literature is kept in a RMS, then potentially interesting articles can be easily put aside by moving the article to a dedicated folder. Alternatively, the reading of the articles might precede the emergence of the RQ. Good practices for arranging read articles is based on folders. A RQ could arise from any folder. Hence, any folder in the RMS could eventually become a RW-amenable folder.

(DP2) **Provide the RMS with** RW-amenable folders **in order for researchers to** seamlessly promote existing folders to become RW variables.

### Open Coding: Moving From RW to RW’

Research is rarely a secluded but a collaborative activity (Ellemers 2021; Marijan and Gotlieb 2021). This might especially be so for PhD projects, mainly during the critical stage of finding the RQ.

(DP3) **Provide the RMS with** sharing facilities **in order for researchers to** unveil their progress to their peers.

Once articles are moved to a RW-amenable folder, coding starts. Coding implies two tasks: data extraction and code assignment. Data extraction results in a set of “excerpts” for each article. Collected excerpts under a theme are next re-read for the codes to emerge. Codes are realized as descriptive labels that directly describe or are taken from the text (aka *In Vivo* coding). Ideally, this set of codes is mutually exclusive and/or well-defined from earlier literature (aka a priori coding). The CGAR template is a case in point. Here, we take *Context, Goal, Artifact* and *Requirements* as the initial codes to label excerpts. In this way, each article in the RW turns into a case in which to spot these four concepts.

To this end, advanced QDA tools resort to color-coding highlighting (e.g., *NVivo*). Here, colors stand for codes (e.g., using red to highlight text fragments related with *Context*), and color-coding highlighting serves to both extract and assign codes to excerpts. Color-coding is one of the most valuable affordances of QDA tools. RQ Scoping sticks to this mechanism.

(DP4) **Provide the RMS with** color-coding highlighting **in order for researchers to** seamlessly conduct coding.

Our setting is not a priori coding, i.e., the coding scheme can be open and emerge during the coding process itself. Excerpts from RW inspire new codes, which, in turn, might lead to reconsider the current RQ, moving from RQ to RQ’, i.e., changing (some of) the (t, c) pairs. This might involve re-coding some of the studies in the RW. The researcher might need to go back to earlier read articles and/or excerpts. Hence, revisiting formerly read articles is an important part of open coding (Ortlipp 2008; Bandara 2006).

(DP5) **Provide the RMS with** excerpt visualizations **in order for researchers to** re-code affected articles.

Better profiling the research question (i.e., moving from RQ to RQ’) might cause a shift in focus on the related work to be considered, i.e., moving from RW to RW’. After all, the significance of the RW is not about size (i.e., the number of articles being considered) but the extent these articles confirm/defy the current RQ. In Glaserian GT, theoretical sampling is a way of collecting data, and deciding what data to collect based on the theory and categories that emerge from the data. In our setting, RW and RQ are the counterparts of the ground data and the theory, respectively. Therefore, our way to make RW evolve can be characterized as “theoretically sampled” insofar as the theory (i.e., RQ) drives the next RW sampling (i.e., RW’). If RW sampling is realized in terms of queries upon bibliographical databases, then these queries are to be constructed out of the current RQ. If the RQ is a set of (t, c) pairs, then the query is an AND connective of (t=c) expressions. Unfortunately, bibliographical databases follow slightly different query languages, hence the Boolean connectives are expressed differently depending on the targeted database. Therefore, Scoping utilities should help with the construction and mapping of RQ-driven queries targeted to potentially more than one bibliographical database.

(DP6) **Provide the RMS with** the ability to query bibliographical databases based on the current RQ **in order for researchers to** effortlessly shift in focus on the related work to be considered.

### Axial Coding: Moving from RQ to RQ’

Glaserian GT follows an inductive approach to theorizing: from transcript data to concepts. Firstly, codes are created through Open Coding and, after, they are grouped by similarity of meaning, originating the first themes (aka categories). During Axial Coding, these first themes might be clustered around more abstract themes, leading to a hierarchy of themes. Our approach is not fully inductive. We start with CGAR as the overarching themes. Confronted with a given RW, the researcher wonders what are the essential (vs. the accidental) characteristics of her own work insofar as providing a new solution to an old problem, or appropriating an existing solution to address a new problem. To better profile one’s work, CGAR themes might need to be refined. In other words, the RQ is in flux. Operationalization wise, this results in a frequent splitting, merging, adding or renaming of the CGAR constructs.

(DP7) **Provide the RMS with** means for splitting, merging, adding or renaming of themes **in order for researchers to** conduct axial coding, and better discriminate one’s work.

“Splitting”, “merging” or “renaming” describe functional requirements but not the quality of how these operations are conducted. In this regard, two issues emerge.

First, Axial Coding is a critical step better framed as a collaborative effort where postgraduate students work in tandem with their supervisors. Comparative Thinking is not a secluded activity. Hence, finding appropriate visualizations turns pivotal to support collaborative Comparative Thinking.

Second, in Agile Literature Reviews the Axial Coding is an activity sustained over time. By contrast, QDA and SLRs tend to be one-shot efforts. They start and eventually come to an end without main disruptions. By contrast, problem-solving ESE encompasses both problem understanding and solution development (e.g., a software artifact). Therefore, RQ Scoping might require a halt in keeping up-to-date with the literature in order to build (and evaluate) the artifact. RQ Scoping is marked by fits and starts rather than a steady progress. Here, resumption utilities come in handy to facilitate a quick recovery of the mindset previous to the interruption.

(DP8) **Provide the RMS with** means for comparative analysis **in order for researchers to** track the progress so far, facilitating Comparative Thinking and a fits-and-starts way to scope the RQ.

### Iterating: The Co-Evolution of RQ and RW

RQ and RW evolve in tandem. To ensure coherence in coding criteria throughout, code-books permit to trace back the process of synthesizing the codes and translating them into themes, improving the soundness of the study (Glaser and Strauss 2017). This role of code-books as coding-decision diaries is even more important in the presence of discontinuity and supervision, both aspects present in our setting. In a fits-and-starts scenario, code-books allow for a prompt resumption as well as a shared understanding with supervisors. In addition, code-books should be version-controlled. This is, “chronological code-books” should track the main reasons behind each of the choices, forcing the researcher to document the motivation or logic for each decision made. From our perspective, chronological code-books help with “memoing”, a term used in GT to denote the process of writing memos (e.g., notes, diagrams or sketches). Together with coding, memos are the primary record of how the researcher has engaged with the data in pursuit of concepts and theory. Memos need to account for how categories and concepts have developed, and in cases where theory has emerged, explain how it has emerged. Hoda (2021, p. 17) highlights memoing as “an imperative procedure that distinguishes STGT from other qualitative research methods”.

(DP9) **Provide the RMS with** chronological code-books **in order for researchers to** support transparency, replication and eventual backtracking.

So far, we introduced a set of design principles that assume that in a given context (i.e., problem-solving research and novice researchers), a given set of affordances (e.g., theoretical sampling) will hopefully accomplish stakeholder goals, (i.e., finding a design question). Table 2 summarizes the outcome. A proof of concept is needed.

**Table 2:** Design principles for RQ scoping

ID	Provide Scoping Utility with...	In order for researchers to...	FRAMEndeley’s realization
DP1	A CGAR template	Set CGAR as the departing	RQ Scoping row
		themes for RQ definition	
DP2	RW-amenable folders	Seamlessly promote existing	Re-use of Mendeley’s folders
		folders to become RW variables	
DP3	Sharing facilities	Unveil progress to their peers	Re-use of Mendeley’s
			group mechanism
DP4	Color-coding highlighting	Conduct Open coding	Extended color-
			coding highlighter
DP5	Excerpt visualizations	conduct re-coding	The Theme Canvas
DP6	Query facilities upon	Effortlessly shift in focus on	Search string
	bibliographical	the related work to be	elaboration + snowballing
	databases	considered	
DP7	Means for splitting,	Conduct Axial coding	Theme rearrangement
	merging, adding or		
	renaming themes		
DP8	Means for comparative	Track the progress so far	The Scoping Canvas
	analysis		+ the Article Canvas
DP9	Chronological code-	Support transparency,	Export Scoping
	books	replication and eventual	Canvas + Code-book
		backtracking	Canvas

## Proof of Concept: FRAMEndeley

This section introduces *FRAMEndeley*, a Scoping utility for Mendeley. Elsevier’s Mendeley is at the very top of the RMS rank (Zaugg et al. 2011), which already enjoyed over 2.5 million users in 2013 (Bonasio 2013)—refer to (Granieri 2019) for a recent survey on RMSs. This huge user base now turns into potential beneficiaries of this research. FRAMEndeley is available for download at Chrome’s Web Store.^4^ At Chrome’s Web Store, it can be found a video with instructions for its pairing with the user’s Mendeley account, and the current number of users (512 at the time of this writing).

**Fig. 2:**
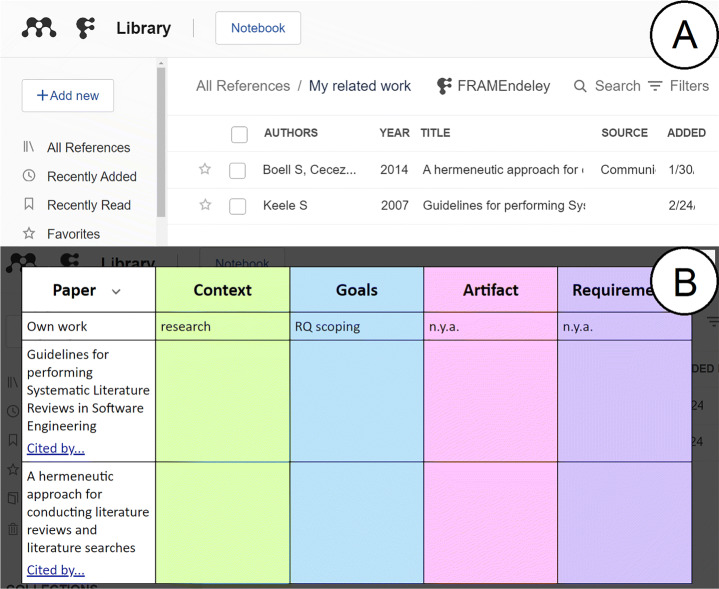
FRAMEndeley button in Mendeley’s library page (A). When clicked, the **Scoping Canvas** pops up (B). At the onset: (1) the RW folder only includes the seminal articles; (2) themes are limited to CGAR, and (3), the RQ might still be unknown for most of the themes

Architecturally, FRAMEndeley is conceived as a layer on top of Mendeley by means of a browser extension (Díaz and Arellano 2015). That is, Mendeley’s web pages are overlaid with a “RQ Scoping layer”. This layer becomes visible when clicking the FRAMEndeley button (see Fig. 2). The use of a browser extension architecture brings some benefits in our setting: installability (i.e., extension installation is commonly limited to a single click), familiarity (i.e., the odds are that students already enjoy some extensions in their browsers), and configurability (i.e., extensions are locally installed; should appropriate configuration parameters be defined, this permits tuning the behavior of the extension to the student/supervisor preferences).

The rest of this section discusses how FRAMEndeley realizes the aforementioned design principles (see Table 2). We follow Bitzer and Janson’s (2014) meta-design principle of technology adoption whereby when extending a tool with a new utility, strive for seamless integration to gain in efficiency and the chances of adoption (i.e., lowering the learning barrier). The importance of sticking to Mendeley’s gestures will be a *leitmotif* throughout this section.

### Initializing the Variables

**RW variables** are fleshed out as Mendeley folders. Any Mendeley folder can become a RW by just clicking the FRAMEndeley button when on the folder. What makes a RW folder different from other folders is the existence of a workspace for coding (hereafter referred to as the “Scoping Canvas”, see Fig. 2). This canvas is depicted as a table: rows stand for the titles of the articles in the folder; columns correspond to themes; a cell *(a, t)* holds the excerpts that characterize article “*a*” with respect to theme “*t*”. Figure 2 displays this canvas at the onset. The important point is that articles’ titles and excerpts are automatically obtained from Mendeley. Indeed, Mendeley and FRAMEndeley are kept in sync so that new articles or additional highlights (i.e., excerpts) are promptly reflected at the Scoping Canvas (see Fig. 4).

**RQ variables** are fleshed out as the first row in the Scoping Canvas. The cells of this row hold the codes that characterize one’s work according to the themes identified so far. For a start, RQ holds {(C,“n.y.a.”), (G,“n.y.a.”), (A,“n.y.a.”), (R,“n.y.a.”)} where “n.y.a.” stands for “not yet available”. Unlike the rest of the rows that are automatically filled up based on the highlights of the articles, the RQ row is directly handled by the researcher.

**Fig. 3:**
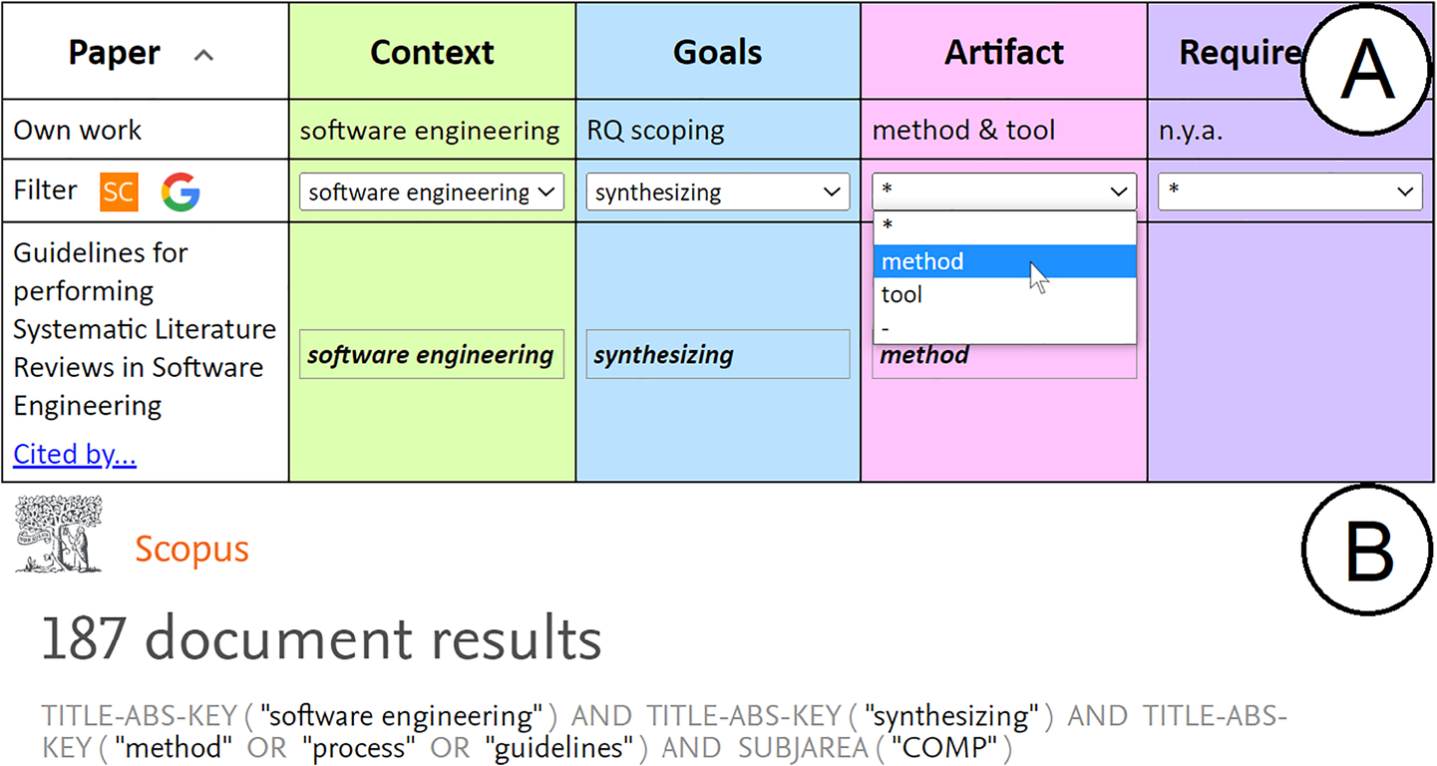
The **Search String row** (A) and the generated Scopus query counterpart (B). For each theme, the researcher chooses a code from the drop-down menu where “*” stands for void. The menu collects the codes identified so far for the theme at hand. The two buttons at the left-hand side serve to launch the query to either Scopus or Google Scholar (A). FRAMEndeley maps it to the query language of the targeted bibliographical database. If a code has different synonyms, as collected in the code-book (see Fig. 8), all the terms are included (e.g., “guidelines” or “process” as synonyms of “method”) (B)

Literature Sampling refers to the process of populating the RW folder. As any other Mendeley folder, articles can be imported from either the researcher’s desktop or directly from the web.^5^ Either way, studies are cherry-picked based on the intuition of the researcher. By contrast, Literature Sampling resorts to the existing RQ to ascertain next sampling. A RQ is a set of pairs *(t, c)*. FRAMEndeley constructs a query string as a conjunction of “*t*=*c*” expressions. This is supported through the *Search String row* (see Fig. 3). The cells of this row are filled up based on the current codes. The resulting expression is next mapped to the syntax of either Scopus or Google Scholar. The query result shows up in a separate browser tab at the Scopus/Google Scholar site. Once there, Mendeley’s Web importer can be used to directly populate the RW folder, or some auxiliary folder where articles are inspected before being moved to RW.

We conducted some evaluations with postgraduate students using FRAMEndeley. They appreciated the systematicity that the Search String row brings about. However, they also requested a search that pivots around articles, i.e., forward snowballing. To this end, we enriched the titles of the articles with a snowballing link: *cited by* (see Fig. 2). Click it for obtaining the list of studies citing this article through Scopus. The outcome is displayed in a separate browser tab. This approach is akin to proposals for augmenting websites with external search services and in-context results display (Bosetti et al. 2022).

### Open Coding

#### Data Extraction

**Fig. 4:**
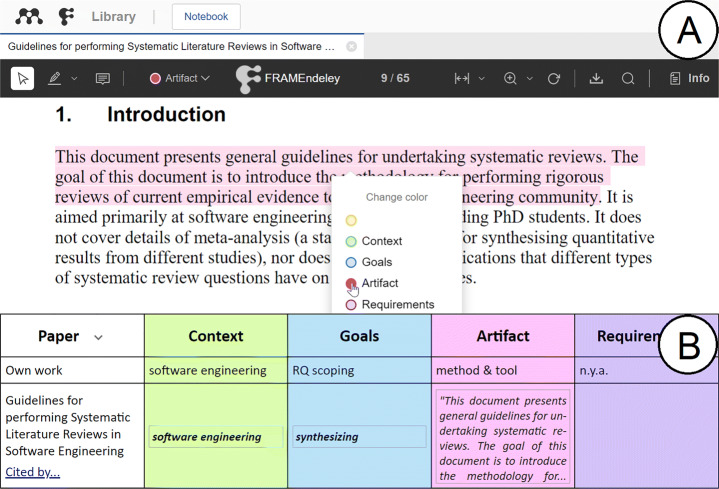
Data Extraction is conducted through color-coding highlighting (A). Notice that the color palette now includes a legend with the code counterpart. So extracted excerpts (e.g., “This document presents general ...”) are initially held as cell values of the Scoping Canvas (B)

FRAMEndeley supports color-coding highlighting. At the onset, CGAR themes are mapped to four colors. Researchers use these colors when highlighting text fragments in articles: highlight in pink for the excerpt to show up in the “Artifact” column (see Fig. 4). Highlighting wise, FRAMEndeley taps into Mendeley’s utilities. The only addition is that Mendeley’s color palette now includes a legend with the code counterpart (see Fig. 4).^6^ Highlighted fragments become excerpts. Initially, excerpts are reflected in the Scoping Canvas as cell values. We are now ready for coding.

#### Data Coding

**Fig. 5:**
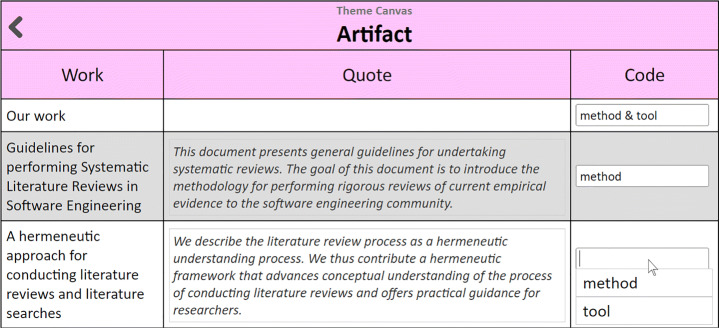
The **Theme Canvas**. This canvas unfolds the excerpts that sustain the characterization of the articles according to their coding. A drop-down menu helps select codes that have been previously introduced. The introduction of a brand-new code requires an additional entry in the code-book (see Fig. 8). Codes can be changed at any time

Initially, there are just excerpts and no codes. As time goes by, codes emerge until the point where excerpts can be mostly pigeonholed along the existing codes, i.e., excerpts are substituted by codes as the cell values. For the sake of coherence and traceability, it is important to keep accessible all excerpts and the coding decisions throughout. To this end, the Scoping Canvas keeps a reservoir of excerpts-by-theme: click a Theme cell (i.e., the headings of the columns) for its Theme Canvas to show up. Figure 5 depicts the case for the “Artifact” theme. This retrieves excerpts-by-theme.

**Fig. 6:**
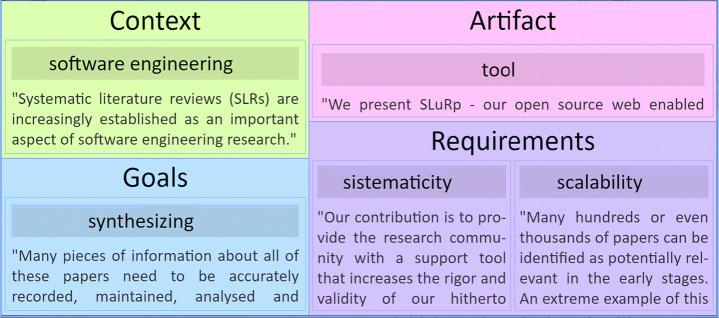
The **Article Canvas**. This canvas outlines how the article is positioned according to the current themes, i.e., how it has been coded and which excerpts sustain this coding. Click a quote to see it in context, i.e., back to the place in the article where this excerpt appears

In addition, the reservoir of excerpts-by-article also comes in handy. Here, the researcher wants to have the overview of the whole article to better ascertain the most adequate codes. This results in the Article Canvas, a layer on top of Mendeley’s document viewer that displays how this article is arranged along the current themes (see Fig. 6). Both the Article Canvas and the Theme Canvas are kept in sync.

### Axial Coding

#### Re-coding

**Fig. 7:**
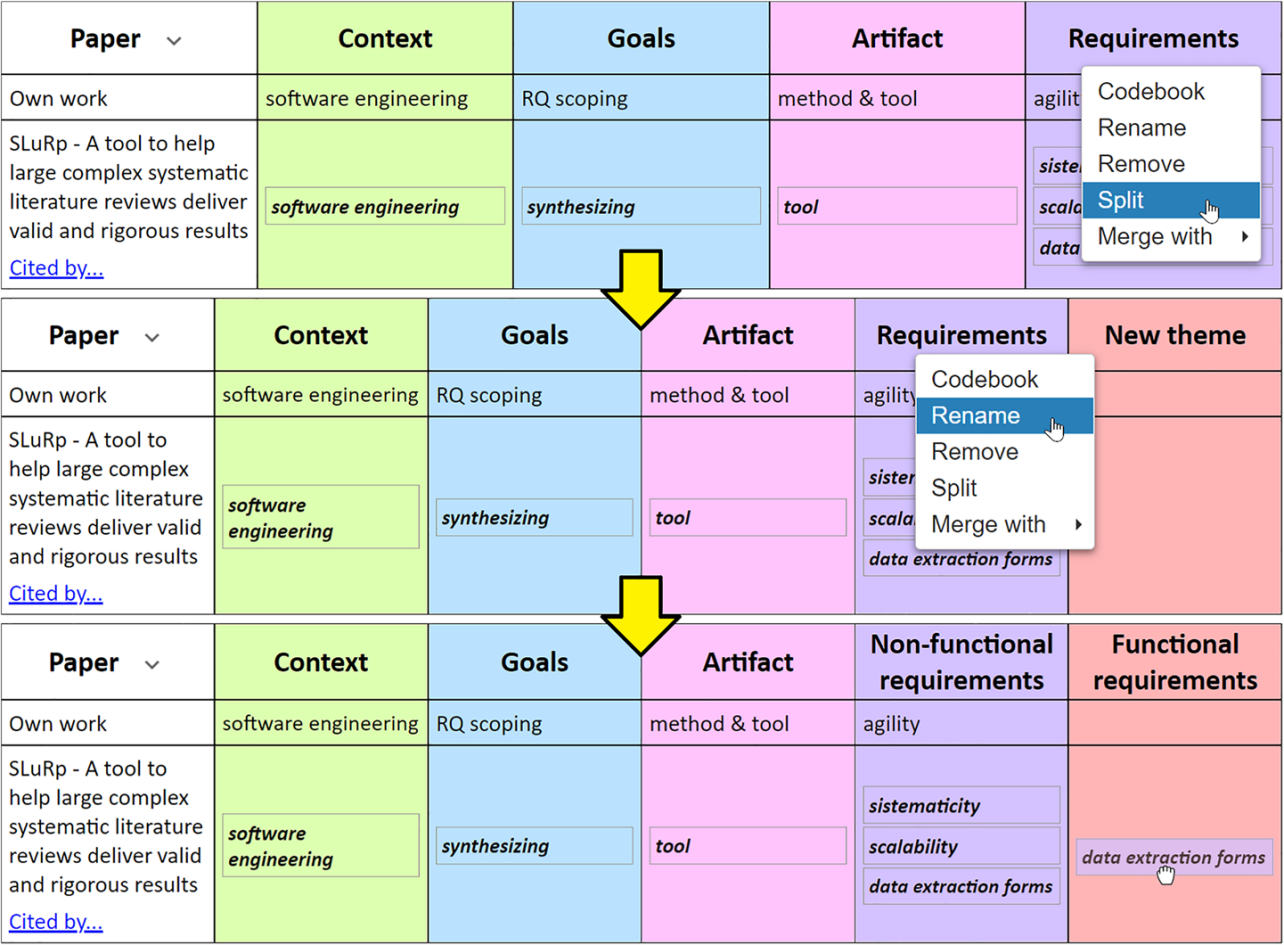
If a theme is split, then a twin theme is created. Existing codes can be either moved to the twin theme or kept at the original theme

Theoretical sampling implies themes/codes to be in flux. At the onset, FRAMEndeley provides the CGAR themes. Throughout the research, these themes are elaborated, i.e. removed, renamed, split (see Fig. 7) or merged, and FRAMEndeley ensures a 1:1 correspondence between colors and themes. Implementation wise, these theme operations are supported through a right-click menu upon the Theme cells.

#### Code-books

**Fig. 8:**
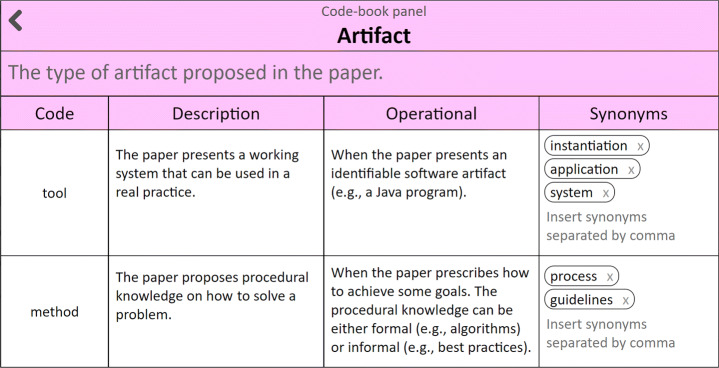
The **Code-book Canvas**. It collects the code label, a descriptive definition, an operational description, and synonyms that come from article excerpts

Code-books sustain coherence throughout the coding process. Boyatzis (1998) recommends for code-books to include the code label, the definition of what the code concerns, and a description about when to use it. FRAMEndeley provides the Code-book Canvas to collect this information as well as synonyms of importance during search string construction (see Fig. 8). This panel pops up every time a new code or theme is introduced. No support is given for naming codes or themes.

### Iterating

#### Resumption

RQ Scoping is marked by fits and starts rather than a steady progress. FRAMEndeley helps moving in and out during RQ Scoping. First, the Scoping Canvas provides an overview of the progress so far: which articles have been coded or how they have been coded. In this way, the Scoping Canvas provides an outline of the RW at a glance. The canvas becomes a sort of map of the research space being investigated. During the research, the extent of the change in the RW is expected to be asymptotic, i.e., frequent shift of articles at the start to be later toned down as the researcher gains more accurate insights about both the problem and the solution.

#### Memoing

FRAMEndeley provides utilities to export the Scoping Canvas in either CSV or LaTeX format. This includes not only the table as such but also the bibliographical references (e.g., a “.bib” file). Table 1 is practically the one produced by FRAMEndeley!

#### Sharing

Mendeley allows for folders and highlights to be shared among users through Mendeley groups. FRAMEndeley taps into this capability by making the Scoping Canvas part of the group sharing. In this way, distinct researchers can work on the same related work and code-book. No new gesture is needed.^7^

Table 2 (third column) summarizes FRAMEndeley’s mechanisms that realize the design principles for Scoping utilities.

## Qualitative Evaluation

We started by arguing about the difficulty in assessing what a good RQ is. This vindicated a shift in focus from the result (i.e., RQs) to the process (i.e., RQ Scoping). Hence, the expected utility of FRAMEndeley should not be judged so much for the goodness of the final RQ but the quality of the process to reach this RQ in partnership with the literature. This “quality” is set in terms of “agility” i.e., the ability to move quickly and easily in the interplay between CGAR-driven thematic elaboration and Literature Reviewing. Therefore, the RQ of this evaluation is:

*what is the role played by FRAMEndeley in promoting “agility” in RQ Scoping?*

This section describes the design, execution and results of a focus group to assess this question, which was conducted following the guidelines in Kontio et al. (2008).

A focus group is a group interview involving a small number of demographically similar participants who have common traits or experiences (Parker and Tritter 2006). In SE, focus groups are proposed as an empirical approach for obtaining qualitative insights and feedback from practitioners, particularly during the early stages of research for identifying problems (Kontio et al. 2008). The group setting allows for the emergence of ideas or opinions that are not usually uncovered in individual interviews.

### Design

We planned a single in-person focus group session with PhD students with an approximate duration of 90-120 minutes. We identified a set of issues to be addressed, and structured them according to the stages of the RQ Scoping process. As highly diverse groups could result in shallow discussion (Bloor et al. 2001), participants were recruited along with the following criteria:

- PhD topic: SE-related
- CGAR entry point: for the participants in the focus group, the RQ challenge rested more on profiling the solution rather than the problem. Supervisors tended to have a clearer picture about what the problem was, and asked the student to look for a solution
- PhD stage: at least one year from the start of their doctoral studies
- Tool setting: use of Mendeley as the RMS as a prerequisite for the use of FRAMEndeley
- FRAMEndeley exposure: at least 4 months of continuous use

We identified 3 potential participants from the University of the Basque Country.^8^ All the participants were between 25 and 31 years old.

### Execution

Due to a last-minute COVID-19 lockdown by one of the participants, the in-person session was reshaped as a hybrid meeting. Thus, the affected participant was able to take part in the session via the Webex videoconferencing software. The focus group lasted for 98 minutes. It was moderated by the second author, while the first author attended the session as an observer and took notes. The session began with a presentation on the goals of the study and an explanation of the RQ Scoping process, which was later employed as a frame of reference to structure the focus group. Before starting with the discussion, participants were asked to fill a form asking them to rank FRAMEndeley’s main mechanisms (see Table 2) in terms of the perceived utility. The audio of the session was recorded using both Webex and a smartphone’s voice recording application. The recording was next transcribed and analyzed by iteratively using FRAMEndeley for open coding, and MindMeister for axial coding.

### Data Analysis and Results

**Fig. 9:**
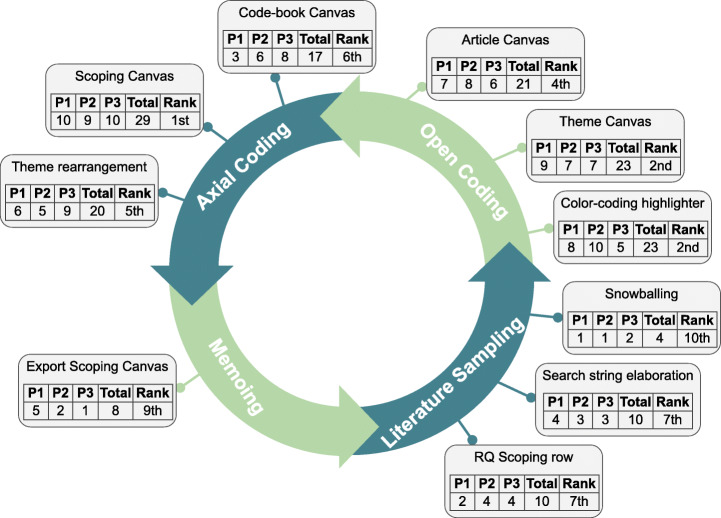
Participants’ perceptions of the utility of FRAMEndeley’s mechanisms organized along with GT’s main activities. For each mechanism, the following information is provided: the participants’ score (P1-P3), the total score and the rank position

At the onset, participants were asked to rank FRAMEndeley’s mechanisms from 1 (least useful) to 10 (most useful). Figure 9 shows the results. At the top, participants place the Scoping Canvas, the Theme Canvas and the extended color-coding highlighter. At the bottom, the snowballing facility and the utility to export the Scoping Canvas. Figure 9 places the results along with “the agile loop”. Broadly, preferences are arranged as follows: *Memoing < Literature Sampling < Axial coding < Open Coding*. Notice that this does not mean that “Memoing” is not important but rather that FRAMEndeley does not add much value to “Memoing” with respect to existing practices. Likewise, students did not appreciate much Literature Sampling mechanisms: “if FRAMEndeley didn’t exist, I would then look for literature directly in Google Scholar. FRAMEndeley reduces some clicks but it is not a great deal insofar as snowballing is concerned”. By contrast, FRAMEndeley shines up for Open Coding and Axial Coding with respect to existing practices basically based on taking notes either manually or using a word processor and arranging ideas using mind maps.

Participants agreed that their journey departed from general articles on the field (usually provided by their supervisors), to later narrow the focus towards a particular problem or an existing solution. When it comes to the start-up effort: “I tried NVivo back in the day and it was a very complete tool, but very difficult to start-up. In this aspect FRAMEndeley is well integrated within Mendeley and very easy to start-up”. Participants highlighted the synergistic integration with Mendeley: “I think that both Mendeley and FRAMEndeley fit rather well. Mendeley helps you to organize your articles and access them easily. ... And next, FRAMEndeley helps you to visualize what you have done so far, to structure it or to conduct searches in the literature”. However, some concerns were raised regarding FRAMEndeley’s dependence upon Mendeley: “if Mendeley makes a change, FRAMEndeley might fall apart”.

The three participants reported the use of FRAMEndeley for exploration purposes, i.e., to elucidate a suitable research path through literature navigation. As one of the students indicated: “we are thinking of using DevOps but I have a very basic knowledge of DevOps. So, what I have done first is to immerse myself in the DevOps literature and what problems or what casuistries are there in that area of DevOps, and then, I have tried to focus a little on the problem that I want to solve, or, better said, the problem that I suspect that may exist”. In addition, two participants highlighted the utility of FRAMEndeley for writing the Related Work section of articles. As one of the students explained: “when it came to writing an article, FRAMEndeley helped arrange related work by focusing on the concepts that make a difference. We had already developed a first solution and we wanted to focus a bit on what the competing options were and how we differed, how we improved, or how our problem setting differed from other studies”. Another recurrent comment was the ease of rearranging themes and moving codes from one theme to another in the Scoping Canvas. Last but not least, the fact of reading with a focus on the CGAR themes was also regarded as beneficial for speeding up reading. Yet, as argued by one of the participants, this can be a double-edged sword: “if themes are preset and I only focus on those ... it could happen that I am overlooking some other aspect that could be important later”. What if you come across an interesting text fragment that cannot be accommodated within the existing themes? All the participants found a workaround by creating a catch-all theme for gathering those interesting ideas that did not fit existing themes. In this regard, participants appreciated the Article Canvas as for the immediacy to remember what an article was about.

When asked about their workflow, participants described an iterative process comprising several small sprints: “most times, I don’t do the coding of a single article, but I highlight some of them, three or so in one go, and then I go to the Theme Canvas and code those three articles, because seeing the comparative helps me to see whether I need to define new codes. ... Before advancing or adding more articles, I see what the previous ones were about”. Besides, participants agreed on the usefulness of having different canvases, and most importantly, on being able to easily shift between them: “navigating between FRAMEndeley’s different canvases is quite fast. ... In general, everything is well integrated and in one or, at most, two clicks you get from one to another”. Another recurrent theme was FRAMEndeley’s fit within participants’ way of understanding research, and especially, its support for collaboration. As one participant said: “in the end, research is essentially collaborative. And sharing the work in the same folder opens the door to having more than one person working on it, and you can even divide the work using the same code-book”. This view was echoed by another participant, who added up: “it comes in handy not only to divide work, but also when you meet [your supervisor] and want to talk about the comparison that you have been doing, the fact that everyone has had access to the analysis and shares the same vocabulary greatly facilitates the discussion”.

Broadly, a workflow pattern seemed to emerge that starts with supervisors pointing out some “seminal readings”. This initial reading led students to come across other articles of interest. The Scoping Canvas seemed to foster Comparative Thinking. Thematic splits tended to be consulted with the supervisors.

### Discussion

Back to the RQ of this evaluation, the role of FRAMEndeley in promoting “agility” seems to rest on two main enablers: the shallow learning curve (partially due to its integration with Mendeley) and the fostering of focus (e.g., low friction in canvas shifting). On the downside, participants worried about the lack of portability and fragility of FRAMEndeley upon Mendeley client-side implementation. An interesting insight was a potential “channel effect” on the CGAR template that might prevent students from thinking laterally, considering concerns other than those tackled by the current themes.

### Threats to Validity

We consider threats for qualitative research as described in (Maxwell 1992).

**Descriptive Validity** refers to the accuracy and completeness of the data. We transcribed the session and took notes about participants’ reactions. Moreover, we asked for clarifications from the participants on the doubtful extracts of the discussion.

**Interpretive Validity** refers to how well researchers capture participants’ meaning of events or behaviors without imposing their own perspective. To improve interpretative validity, we began the session with a brief presentation on the objectives of the study and the proposed intervention. This presentation was aimed at establishing a common terminology and avoiding misunderstandings. Apart from that, we confirmed the conclusions of the focus group by soliciting feedback from participants. Moreover, the transcript was translated from Spanish to English, but the data analysis was made with the original Spanish version so as not to alter participants’ intended meaning.

**Theoretical Validity** refers to the extent to which researchers provide an accurate and coherent explanation of the phenomena. The main threat to theoretical validity is concerned with the thematic analysis of the data. We tried to mitigate this threat by carrying out several iterations of coding, followed by discussion among the two authors of the article.

**Generalizability** is concerned with the applicability of conclusions to similar settings and groups (i.e., internal generalizability) (Maxwell 1992). To this end, we profile our subjects in terms of PhD topic, CGAR entry-point, PhD state, tool setting and FRAMEndeley exposure, to capture relevant contextual variables. Yet, other contextual factors might exist that have not been noticed here. All in all, the sample is too low and the population too complex to make any claim of generalization. Next section extends the scope to a larger population but at the expense of looking at “usage intention” rather than “actual usage”.

## Quantitative Evaluation

This section reports an evaluation based on the Unified Theory of Acceptance and Use of Technology (UTAUT), one of the most frequently used models of IS/IT adoption (Venkatesh et al. 2003). This model encompasses four main constructs that determine usage intention: performance expectancy, effort expectancy, social influence, and facilitating conditions. The latter two constructs are especially convenient for our purposes since the use of ITDT (and eventually FRAMEndeley) is conditioned by the dominant culture on literature reviewing in the research department. The aim of this study is then

*to understand the factors contributing to the adoption of FRAMEndeley by postgraduate students.*

### Hypotheses

**Performance expectancy** is defined as “the degree to which an individual believes that using the system will help him or her to attain gains in job performance” (Venkatesh et al. 2003, p. 447). Here, the job is RQ Scoping. Hence, performance expectancy refers to students’ belief that using FRAMEndeley will help better scope their RQ. Different studies underline the disorientation of students when consulting the literature (Klopper et al. 2007; Ruben 2016). The expectation is that FRAMEndeley would favor a more productive and performant reading when conducting RQ Scoping. Therefore, we posit that:

H1: Performance expectancy has a positive effect on students’ behavioral intention to use FRAMEndeley.

**Effort expectancy** is defined as “the degree of ease associated with the use of the system” (Venkatesh et al. 2003, p. 450). The expectation is that by seamlessly embedding ITDT within a RMS, the effort of conducting RQ Scoping will be reduced. Indeed, theories on the role of structural embeddedness of technology during the learning process recommend new tasks (e.g., RQ Scoping) to be embedded in the place where users already conduct related tasks (e.g., reading) (Gupta and Bostrom 2013). This leads to gains in both effectiveness and the chances of adoption (Bitzer and Janson 2014). Accordingly, the following hypothesis is postulated:

H2: Effort expectancy has a positive effect on students’ behavioral intention to use FRAMEndeley.

**Social influence** is defined as “the degree to which an individual perceives that important others believe he or she should use the new system” (Venkatesh et al.^2003^, p. 451). The current study considers the social influence of supervisors, fellow postgraduate students, and academic administrators that might encourage the use of ITDT as part of their research methodologies. Consequently, this study proposes that:

H3: Social influence has a positive effect on students’ behavioral intention to use FRAMEndeley.

**Facilitating conditions** is defined as “the degree to which an individual believes that organizational and technical infrastructure exist to support use of the system” (Venkatesh et al. 2003, p. 453). For our purposes, facilitating conditions are considered as the accessibility of an appropriate learning environment (e.g., courses about Thematic Analysis) and infrastructure within the university (e.g., RMS adoption, support for installing browser extensions) that can foster the use of FRAMEndeley. The relationships between facilitating conditions and behavioral intention were statistically significant as noted by earlier scholars (Chauhan and Jaiswal 2016). Hence, the following hypothesis is formulated:

H4: Facilitating conditions have a positive effect on students’ behavioral intention to use FRAMEndeley.

### Design

#### Sampling and Population

A quantitative approach was employed to gather the data for the study. Data was collected through a questionnaire on completion of a 20-hour course on Literature Review at the National University of the Northeast in Argentina. As part of this course, one of the activities was the use of FRAMEndeley to unleash Comparative Thinking based on a set of studies. The activity was compulsory for all the participants of the course. A total of 31 students participated in the study, of whom 11 were discarded for being undergraduate students.

Although all the participants were postgraduate students, the sample was diverse. The age of the participants ranged from 25 to 60 (mean: 36.6; std. dev.: 10.11), and nearly two thirds were female. With respect to their educational level, 55% of the sample were pursuing a MSc (n = 11/20) and the remaining 45% a PhD (n = 9/20). In the latter case, participants also differed in the stage of their doctorate: 22.2% (n = 2/9) were in their first year, 11.1% (n = 1/9) in their second, 22.2% (2/9) in their third and the remaining 44.4% (4/9) were in their fourth year and beyond.

#### Questionnaire Development

The questionnaire contained two separate sections. Section A requested socio-demographic information. Section B examined the factors affecting students’ behavior along with measurement items proposed in the UTAUT model (Venkatesh et al. 2003). A Spanish version of the questionnaire was used with minor textual adaptations whereby general terms in the original version were replaced to apply to the specific context of the evaluation (e.g., “the system” was rephrased as “FRAMEndeley”) (Madera et al. 2012). Section B comprised 16 items measured on a five-point Likert scale ranging from 1 (strongly disagree) to 5 (strongly agree).

### Data Analysis and Results

The Partial Least Square-Structural Equation Modeling (PLS-SEM) technique was employed to estimate the effects among the hypothesized constructs (Russo and Stol 2021). SmartPLS software (version 3.3) was used to test the research model through a two-step data analysis: measurement model and structural model.

#### Measurement Model

The first step of the process was to endorse the reliability and validity of the measures. The internal consistency and reliability of the constructs were assessed using Cronbach’s *α* and composite reliability (CR). Table 3 shows that all composite reliabilities and all but one Cronbach’s *α* topped 0.70.^9^ According to Hair et al. (2010), this can be considered an acceptable threshold. Therefore, the results confirmed the reliability of the measures.

**Table 3:** Reliability and factor loadings

ID	Items	CFA	Cronbach’s	CR	AVE
		loadings	alpha		
	Performance expectancy (PE)		0.669	0.808	0.584
PE01	I would find FRAMEndeley useful	0.775			
	throughout my postgrad studies				
PE02	Using FRAMEndeley enables me to keep focused while reading	0.738			
PE03	Using FRAMEndeley increases my productivity	0.779			
	Effort expectancy (EE)		0.799	0.881	0.712
EE01	My interaction with FRAMEndeley would be clear and understandable	0.823			
EE02	It would be easy for me to become skillful at using FRAMEndeley	0.834			
EE03	I would find FRAMEndeley easy to use	0.874			
	Social influence (SI)		0.870	0.920	0.795
SI01	People who influence my research practice think that I should use FRAMEndeley	0.871			
SI02	People who are important to me, academically wise, think that I should use FRAMEndeley	0.963			
SI03	In general, the organization has supported the use of FRAMEndeley	0.836			
	Facilitating conditions (FC)		0.782	0.873	0.696
FC01	I have the knowledge necessary to use FRAMEndeley	0.786			
FC02	FRAMEndeley is compatible with other systems I use	0.828			
FC03	A specific person (or group) is available for assistance with FRAMEndeley difficulties	0.885			
	Behavioral intention (BI)		0.827	0.881	0.651
BI01	I predict I would use FRAMEndeley in the next 12 months	0.920			
BI02	I plan to use FRAMEndeley in the next 12 months	0.841			
BI03	Should FRAMEndeley not be available for my favorite Web browser, I would be willing to move to Google Chrome for FRAMEndeley sake	0.710			
BI04	Should FRAMEndeley not be available for my favorite RMS, I would not mind to move to Mendeley for FRAMEndeley sake	0.740			

Convergent validity was evaluated by examining the standardized factor loadings and average variance extracted (AVE). According to Fornell and Larcker (1981) and Hair et al. (2010), convergent validity is verified when (1) all measurement items top 0.70, (2) composite reliability is greater than 0.70, and (3), AVE is above 0.50. Table 3 shows that all these requirements were achieved, thereby confirming the convergent validity of the model.

Discriminant validity was examined through the Fornell-Lacker criterion, by comparing the square roots of the AVEs with the correlations among constructs to ensure that each factor was different, or uncorrelated. Table 4 shows that all shared variances between factors in the model were below the square root of the AVEs, indicating that the constructs were distinct from one another.

**Table 4:** Inter-construct correlations

Variables	BI	EE	FC	PE	SI
Behavioral intention (BI)	**0.807**				
Effort expectancy (EE)	0.700	**0.844**			
Facilitating conditions (FC)	0.565	0.568	**0.834**		
Performance expectancy (PE)	0.695	0.694	0.239	**0.764**	
Social influence (SI)	0.285	0.279	0.454	0.314	**0.891**

Bold font stands for the square roots of the AVEs

#### Structural Model

**Fig. 10:**
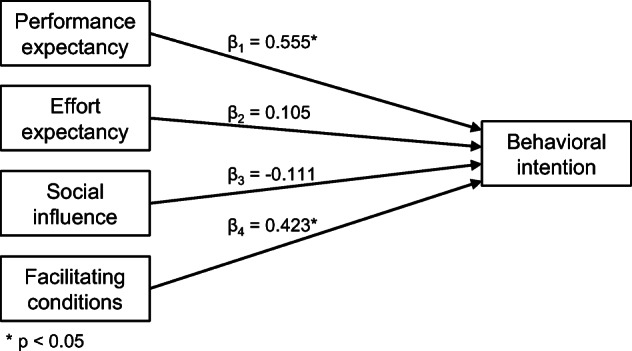
Results of the structural model

In the second step of the PLS-SEM approach, i.e., the structural model, the relationships between the latent variables were examined. Table 5 presents the standardized beta coefficients and t-values of the hypothesized model. The four primary constructs of the UTAUT model (i.e. performance expectancy, effort expectancy, social influence and facilitating conditions) were defined as independent variables, while behavioral intention was specified as the dependent variable in the model (see Fig. 10). The R-square value of the behavioral intention was 0.667, demonstrating that the independent variables accounted for 66% of the total variance in students’ behavioral intention to use FRAMEndeley. These results indicate an acceptable model fit to the data.

Particularly, H1 examined the relationship between performance expectancy and students’ behavioral intention to use FRAMEndeley. Table 5 reveals that performance expectancy had a significant positive effect on students’ behavioral intention in this regard (*β*_1_ = 0.555, t-value = 2.091, p < 0.05). Hence, H1 was sustained.

**Table 5:** Relationships on students’ behavioral intention to use FRAMEndeley

	Hypothesized paths	Estimate	S.E.	t-value
H1	Performance expectancy → behavioral intention	0.555*	0.059	2.091
H2	Effort expectancy → behavioral intention	0.105	0.014	0.366
H3	Social influence → behavioral intention	− 0.111	0.038	0.646
H4	Facilitating conditions → behavioral intention	0.423*	0.044	2.133

*p < 0.05; S.E. = Standard Error

Next, H2 and H3 determined whether students’ behavioral intention to use FRAMEndeley was significantly affected by effort expectancy and social influence, respectively. In this respect, the PLS-SEM findings (*β*_2_ = 0.105, p > 0.05 and *β*_3_ = − 0.111, p > 0.05) showed that both factors had insufficient impact on students’ behavioral intention. Thus, both H2 and H3 were rejected.

Finally, H4 tested whether facilitating conditions had a significant impact on students’ behavioral intention to use FRAMEndeley. The results show that facilitating conditions were a significant contributing predictor of students’ behavioral intention (*β*_4_ = 0.423, t-value = 2.133, p < 0.05). Therefore, H4 was endorsed.

### Discussion

Back to this evaluation’s RQ as for the factors contributing to the adoption of FRAMEndeley, results for behavioral intention are encouraging. Both questions BI01 and BI02 seem to suggest that subjects perceived utility in FRAMEndeley. In this respect, BI03 and BI04 are especially enlightening. Despite performance expectancy (H1), ample adoption might still require ITDT to be framed at the users’ existing tools (e.g., their favorite browser or RMS). This suggests that embedding ITDT as part of the RMS rather than as a dedicated tool *à la NVivo*, is a step in the right direction.

The evaluation does not provide any conclusion as for the impact of facilitating conditions (H2) and social influence (H3). The fact that FRAMEndeley is a browser extension, and hence, most students are familiar with this practice explains H2. As for H3, we conjecture that the unawareness of ITDT among the SE community might explain that not only students but also their supervisors might ignore this method, and hence, we cannot pretend they “support the use of FRAMEndeley”.

Finally, H4 insists on the importance of a proper setting to conduct ITDT. Unlike the traditional use of GT in Social Sciences, ITDT is a continuous effort. From the onset, and well into the project progress, researchers need to place their insights and solutions within a flux of articles while periodically shifting to artifact development and evaluation. In this back-and-forth setting, knowledge (FC01), tool compatibility (FC02) and assistance (FC03) are a necessary scaffold.

### Threats to Validity

For experimentation in SE, we consider here the threats reported in Wohlin et al. (2012). The use of a well-documented instrument (i.e., the UTAUT model) accounts for reliability. There is abundant documentation and case studies that facilitate the evaluation to be consistently replicated. In addition, the UTAUT model is reckoned to have a reasonable construct validity (i.e., UTAUT questionnaire has been validated) and internal validity (i.e., UTAUT constructs have an impact on behavioral intention). Specifically, validation by Venkatesh et al. (2003) of UTAUT in a longitudinal study found it to account for 70% of the variance in behavioral intention to use and about 50% in actual use. In addition, the sample task proposed as part of the course was much simpler than real-world RQ Scoping. Indeed, we characterize RQ Scoping as a continuous effort, but this continuity has not been considered in the evaluation. As for external validity, the results were obtained for postgraduate students that voluntarily attended a course on literature review. This clear interest in the topic might not be shared by other postgraduate students, and hence, limits the generalizability of results. As for the age moderator, the youth of the subjects might make them more positive about using digital tools with respect to more senior researchers.

This study has several limitations. First, this study was conducted within a short period. Students’ perceptions of UTAUT constructs can change over time as new knowledge and experiences are accumulated. Therefore, future studies could employ a longitudinal design to obtain more accurate findings in a more realistic scenario. Second, although social influence is a UTAUT construct, the importance of the student’s supervisor advises to capture this factor as a separated moderator in future studies. Third, results are based on participants’ perceptions and impressions after “playing around” with the tool. This may reduce the soundness of the results. Finally, the number of participants in the study was limited (N = 20). We do not aim at a conclusive evaluation of what is an explorative study. The novelty of the tool, the context diversity where RQ Scoping is conducted, and the continuous nature of RQ Scoping, call for varied longitudinal studies that reach out to distinct research groups.

**Fig. 11:**
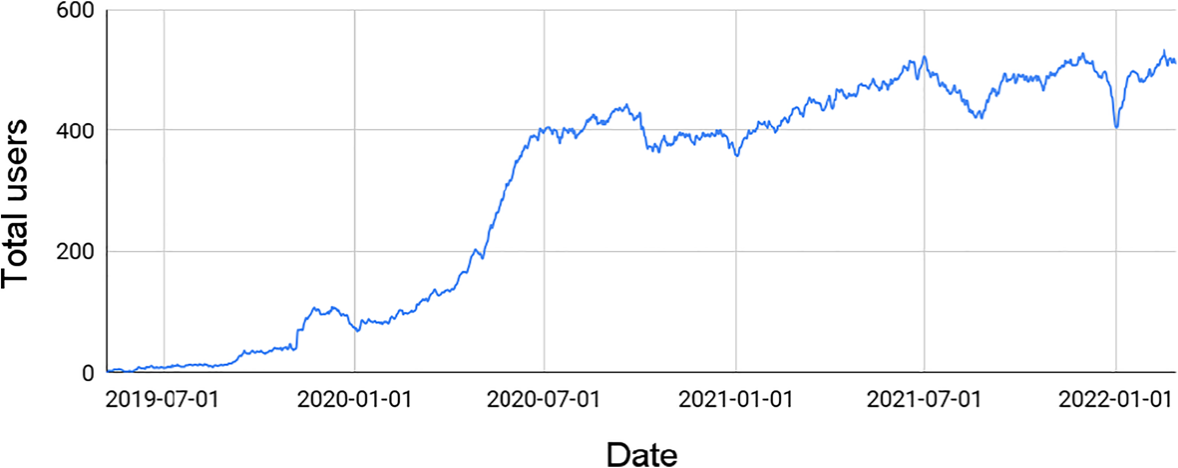
FRAMEndeley’s user base over time. Source of data: Chrome’s Web Store

As a first step, we tracked whether subjects kept using FRAMEndeley once the evaluation was over. To this end, we uploaded *FRAMEndeley* (and one video) to Chrome’s Web Store and we waited to see whether participants found *FRAMEndeley* useful to the extent of installing it. As of June 2022, more than two years after the evaluation took place, *FRAMEndeley* installations scale up to 500 users. Figure 11 depicts the evolution in numbers, directly obtained from Google. The important point is not so much the current number of users but the steady growth with no remarkable declines. This seems to indicate a continuous use of FRAMEndeley.

In this trial, “software installation” is regarded as a proxy for utility. It can be argued that the discretionary effort of installing *FRAMEndeley* provides evidence of enough “perceived utility”. Moreover, the fact that the number of users has remained steady for more than two years points to sustained interest as evidence of utility. However, the main threat to construct validity of this evidence is to interpret “installation” as “real use”. While using Google as a data source provides credibility, it goes with the penalty of not having direct access to‘the users.

## Conclusions

This work tackles a problematic phenomenon (i.e., coming up with a Research Question) by introducing an intervention (i.e., Scoping utilities), especially for a given target audience (i.e., postgraduate students). We now revise the contributions stated at the introduction. First, we introduce RQ Scoping as a problematic practice, specifically in problem-solving ESE, and make a case for the use of ITDT as a promising methodology. Second, we elaborate on design principles for tools that support this practice, advocating for RQ Scoping to be framed within RMSs. We provide a proof of concept through FRAMEndeley. Third, given the exploratory nature of this research, we follow a mixed methods approach to evaluation. First, we resort to a *qualitative* evaluation through a focus group (N = 3). Next, we move to a *quantitative* evaluation through a UTAUT questionnaire (N = 20).

Results from the focus group suggest that the “agility” of FRAMEndeley rests on two main enablers: the shallow learning curve (partially due to its integration with Mendeley) and the fostering of focus (e.g., low friction in canvas shifting). As for the UTAUT evaluation, “perceived utility” and “facilitating conditions” are regarded as the main drivers for the intention to use FRAMEndeley. As for “effort expectancy” and “social influence”, no conclusion could be drawn. The drawback that “intention to use” does not necessarily imply “real use” was lessened by reporting data from Chrome’s Web Store where the FRAMEndeley extension enjoys over 500 installations at the time of this writing.

Future work includes a detailed evaluation of the tool as a whole but also for each of the mechanisms that FRAMEndeley puts together (e.g., color-coding, built-in query construction, etc.). All in all, FRAMEndeley “just” helps with the organization and tracking of excerpts and related work. Yet, the selection of themes and codes, and the Comparative Thinking this involves, is within the realm of human ingenuity. This moves to the foreground “social influence” and the role of the research community to discuss the interest of “Agile Literature Reviewing” to complement “Systematic Literature Reviewing”, specifically for novice researchers.

## Data Availability

https://github.com/JeremiasPerez/FRAMEndeley-Project

## Footnotes

1 We note that CGAR is not far from the PICOC template which is commonly used for RQs in secondary studies (Kitchenham and Charters 2007). PICOC stands for the criteria Population (e.g., application area or specific groups of people), Intervention (e.g., the method of interest), Comparison (e.g., the benchmark), Outcomes (e.g., improvements) and Context (i.e., a description of the setting in which the comparison takes place).

2 Refer to Evers (2018) and Al-Zubidy and Carver (2019) for a survey on QDA tools and SLR tools, respectively.

3 In a recent survey among 102 subjects, Al-Zubidy and Carver (2019) report that features somehow supporting theoretical sampling ranked at the lowest: “support for search process” (2 votes) and “notification of new papers” (1 vote). By contrast, at the top rank “search multiple databases in standardized query” (48 votes), “removing duplicates” (13 votes) and “merging results from Digital Libraries” (6), all concerns pointing to comprehensiveness as the goal pursued.

4 https://chrome.google.com/webstore/detail/decpeaebklmmgfhnnhggeikfhhlbcjpf

5 https://www.mendeley.com/reference-management/web-importer

6 Mendeley provides eight different colors for highlighting. Yellow is used for non-thematic highlighting. This leaves FRAMEndeley with seven colors, i.e., seven themes.

7 Implementation wise, code-books are realized as JSON files. These files are not locally kept at the browser but shared through Mendeley groups. This turns the Scoping Canvas into a collaborative endeavor: the JSON structure is centrally kept at Mendeley’s server while the rendering is conducted at client side through FRAMEndeley.

8 The size of a focus group typically varies from 3 to 12 participants (Kontio et al. 2008).

9 The Cronbach’s *α* for Performance Expectancy (0.669) is slightly below this threshold.
